# Tobacco Use and Mass Media Utilization in Sub-Saharan Africa

**DOI:** 10.1371/journal.pone.0117219

**Published:** 2015-02-23

**Authors:** Thomas N. O. Achia

**Affiliations:** 1 Division of Epidemiology and Biostatistics, School of Public Health, University of Witwatersrand, Johannesburg, South Africa; 2 School of Public Health, University of the Western Cape, Bellville, South Africa; The University of Auckland, NEW ZEALAND

## Abstract

**Background:**

Media utilization has been identified as an important determinant of tobacco use. We examined the association between self-reported tobacco use and frequency of mass media utilization by women and men in nine low-to middle-income sub-Saharan African countries.

**Methodology/Principal Findings:**

Data for the study came from Demographic and Health Surveys conducted in Burkina Faso, Ethiopia, Liberia, Lesotho, Malawi, Swaziland, Uganda, Zambia and Zimbabwe over the period 2006–2011. Each survey population was a cross-sectional sample of women aged 15–49 years and men aged 15–59 years, with information on tobacco use and media access being obtained by face-to-face interviews. An index of media utilization was constructed based on responses to questions on the frequency of reading newspapers, frequency of watching television and frequency of listening to the radio. Demographic and socioeconomic variables were considered as potentially confounding covariates. Logistic regression models with country and cluster specific random effects were estimated for the pooled data.

**Results:**

The risk of cigarette smoking increased with greater utilization to mass media. The use of smokeless tobacco and tobacco use in general declined with greater utilization to mass media. The risk of tobacco use was 5% lower in women with high media utilization compared to those with low media utilization [Adjusted Odds Ratio (AOR) = 0.95, 95% confidence interval (CI):0.82–1.00]. Men with a high media utilization were 21% less likely to use tobacco compared to those with low media utilization [AOR = 0.79, 95%CI = 0.73–0.85]. In the male sample, tobacco use also declined with the increased frequency of reading newspapers (or magazines), listening to radio and watching television.

**Conclusions:**

Mass media campaigns, conducted in the context of comprehensive tobacco control programmes, can reduce the prevalence of tobacco smoking in sub-Saharan Africa. The reach, intensity, duration and type of messages are important aspects of the campaigns but need to also address all forms of tobacco use.

## Introduction

Tobacco use occurs throughout the world and is accompanied by a host of diseases that threaten the health and shorten the life of the user [1–6]. An estimated 1.3 billion people worldwide are smokers; the majority of whom started smoking in adolescence and continued throughout their life. It is further estimated that half of the world’s smokers will lose their life through tobacco related disease by the time they reach middle age. Tobacco related diseases currently kill approximately 5 million people per year, and if unabated, will increase to 10 million deaths each year by the year 2020. An estimated 70% of these deaths will be in developing countries [7].

Studies have suggested a decline in the use of cigarette and other tobacco products in high-income countries. There is, at the same time, evidence of a marked growth in cigarette use in middle and low-income countries [8–10]. Despite this increase occurring primarily among men, there is an upsurge in marketing strategies that target women in sub-Saharan Africa (SSA) [11–13]. These strategies are an attempt to capitalise on the global empowerment drive that has led to improved socioeconomic and sociocultural conditions for women. Increased tobacco use within Sub-Saharan Africa differs from other regions of the world in that it is still in its early stages. Globally, the highest rates of tobacco use and tobacco-related deaths are observed in America, with Africa having the lowest rates of tobacco-related deaths: below 5% among men and below 1% among women [14].

In the past two decades, numerous studies have been conducted with the express aim of identifying the key determinants of smoking and tobacco use [15–25]. Tobacco dependence and the associated difficulties of quitting are blamed on several psychosocial, environmental, and biological factors plus the pharmacological effects of nicotine [26], a substance that is now widely recognized as being highly addictive [27]. Environmental influences, including peer and familial influences have been identified as the strongest contributors in determining how and when cigarette experimentation occurs amongst young people. Additionally, there is growing support in the literature confirming the hypothesis that genetic influences also underlie the initiation and lifetime use of tobacco [28–31].

The WHO Framework Convention on Tobacco Control (WHO FCTC) was a worldwide response to the globalisation of the tobacco epidemic [32]. The FCTC requires State Parties to adopt and implement tobacco control measures, including but not limited to the following: packaging and health warning labels on tobacco products; bans on tobacco advertising, promotion and sponsorship; measures to protect people from tobacco smoke; tobacco tax and price increases; regulation of the contents of tobacco products; regulation of tobacco product disclosure; support for economically viable alternatives; measures to curb illicit trade in tobacco products; liability provisions and others [33]. Despite existing bans on advertisements, young and old people in low and mid-income countries continue to be exposed to tobacco products through the media such as movies, the internet, and magazines which are imported from other countries [10].

The role of mass media in fuelling or deterring the upsurge in tobacco use has been extensively investigated within the context of developed countries [34–37]. Within these settings there is evidence to suggest a rise in tobacco usage with increasing media utilization [14]. Individuals in developed countries have better access to mass media and education compared to their counterparts in low and middle-income countries.

The role played by the utilization of the mass media in driving or curbing the tobacco epidemic in low and middle-income countries, especially SSA, has had limited coverage in the literature. In this study, we used data from the Demographic and Health Surveys conducted in 9 African countries over the period 2006–2011 to provide estimates of the magnitude and distribution of tobacco use by men and women in the sub-Saharan African region. The study also interrogates the complex relationship that exists between tobacco use and media use in these sub Saharan African (SSA) countries, adjusting for socioeconomic and demographic factors.

## Methods

### Ethical considerations

This study was based on secondary data with all participant identifiers removed. Survey procedures and instruments were approved by the Scientific and Ethical Review Committee of Medical Research Institutes in each of the countries, and by the Ethics Committee of the Opinion Research Corporation Macro International Incorporated (ORC Macro Inc.), Calverton, USA. Ethical permission for use of the data in the present study was obtained from ORC Macro Inc. Details concerning the data-collection protocols are available on the Measures Demographic and Health Surveys (DHS) website (http://www.measuredhs.com).

### Data Source

The data for this study came from Demographic and Health Surveys (DHS) conducted in 9 SSA countries between 2004 and 2011 [38–46]. The countries selected were Burkina Faso, Ethiopia, Liberia, Lesotho, Malawi, Swaziland, Uganda, Zambia and Zimbabwe. While DHS surveys have been conducted in more than 80 countries, only in these 9 countries were the respondents asked about their experiences of both tobacco use and media utilization. The target population in each survey women aged 15–49 years and men aged 15–59 years. The data used in this study is available, on request, from the Measures Demographic and Health Surveys (DHS) website (http://www.measuredhs.com).

### Sampling Plan

The DHS employs a multistage stratified design with probabilistic sampling with each household having an equal probability of selection. Every survey was stratified by urban and rural status and additionally by country-specific geographic or administrative regions. Detailed sampling plans are available from survey final reports [38–46].

### Study population and sample size

The study population consisted of women aged 15–49 years (n = 101,316) and men aged 15–59 years (n = 58,146) from 76,131 households in 4119 clusters within the 9 countries. Of the 9 countries, 4 were selected on the basis of having imposed bans on tobacco advertising, promotion and sponsorship in at least 4 of the following 7 areas: national television and radio; international television and radio; local newspapers; international newspapers; billboards; points of sale; and the internet. These 4 countries were Burkina Faso, Ethiopia, Lesotho and Swaziland. The other countries considered (Liberia, Malawi, Uganda, Zambia, and Zimbabwe) had no bans imposed on the advertising, promotion and distribution of tobacco [33].

### Variables

The response variables

The primary questions used to construct the response variables of interest required the respondent to indicate whether they currently: used tobacco (in any form); smoked cigarettes; and used snuff (or smokeless tobacco). Based on these variables, 3 dichotomous response variables (*Smoking status, cigarette smoking status and use of smokeless tobacco status*) were created. Each response variable took the value 1 if a respondent used the form of tobacco in the question, or 0 otherwise.

### Indicator of mass media utilization

Media utilization indices were constructed for both the male and female samples using principal component factor analysis [47]. Three media utilization questions were posed to the respondents concerning: *frequency of reading newspapers or magazines*; *frequency of listening to the radio*; and *frequency of watching television*. To each of these questions respondents gave scores of 0 if ‘not at all’; 1 if ‘*less than once a week*’; 2 if ‘*at least once a week*’ and 3 if ‘*almost every day*’. Each media utilization question was assigned a weight or factor score generated through principal components’ analysis. The resulting media utilization score was then standardised in relation to a standard normal distribution with a mean of zero and a standard deviation of one. These standardised scores are then used to create the break points that define *media utilization tertiles* as Lowest, Medium, and High. For both samples, the three media utilization items yielded a single factor (Table 1). In the female sample, the factor solution explained 57% of the total variance, while the factor solution in the male sample explained 55% of the total variance. The reliability of the media utilization index was accessed using Cronbach’s alpha [48] statistics for both the female (Cronbach’s alpha = 0.77, M = 4.55, SD = 2.96) and male (Cronbach’s alpha = 0.82, M = 5.42, SD = 3.03) samples.

**Table 1:** Principal component factor analysis of media utilization.

	**Female sample**	**Male sample**
**Item**	**Factor 1**	**Factor 1**
**Frequency of reading newspapers or magazines**	0.78	0.78
**Frequency of listening to radio**	0.67	0.68
**Frequency of watching television**	0.76	0.77
**Eigenvalues**	1.64	1.66
**Variance explained**	54.75	55.44
**Cronbach’s alpha**	0.77	0.78

### Other covariates

The other covariates included in the analysis were respondent’s age, region of residence, type of place of residence (rural or urban), religion, education, occupation and socioeconomic status.

### Analysis approach

A bivariate cross tabulation was carried out and rates of tobacco utilization were calculated for each category of the covariates (media utilization, age, region, place of residence, education, religion, occupation and socioeconomic status. Pearson’s Chi-square statistic was used to test for bivariate association between the outcomes of interest and the covariates identified.

Generalized linear mixed effects models (GLMMs) with logit link functions and country specific random effects were fitted to the male and female datasets separately using the *melogit* command in STATA [49]. Five models were fitted to each of the data sets. Model **1** was a GLMM with the media utilization index as the primary covariate. Model **2, 3** and **4** were GLMMs with frequency of reading newspapers/magazines, frequency of listening to radio, and frequency of watching television, respectively, as primary covariates. Finally, Model **5** was a GLMM with frequency of listening to radio, and frequency of watching television, along with the other possible confounding covariates included.

## Results

### Summary analysis

Table 2 presents the results of the bivariate analysis. The prevalence of tobacco use was 20.0% and 3.1% in the male and female samples, respectively. Prevalence of cigarette smoking in the male sample was 18% and 1% in the women sample. The prevalence of snuff use in the female sample, (1.6%), was marginally higher than in the male sample (1.1%).

**Table 2:** Prevalence rates of tobacco use, cigarette smoking and smokeless tobacco use by selected covariates.

	Female				Male			
	*Uses tobacco*	*Cigarette smoking*	*Smokeless tobacco use*	*Total*	*Uses tobacco*	*Smokes cigarettes*	*Smokeless tobacco use*	
	(%)	(%)	(%)	N (%)	(%)	(%)	(%)	N (%)
***Media utilization index***								
low	4.8	0.6	2.2	30674(35.5)	25.0	21.4	1.8	16429(32.5)
mid	4.6	0.4	2.8	26906(31.1)	23.0	21.5	1.4	17111(33.9)
high	1.6	0.4	0.9	28853(33.4)	15.8	15.0	0.6	16940(33.6)
***Frequency of reading newspaper or magazine***								
Not at all	4.4	0.5	2.3	62943(72.8)	24.1	21.5	1.6	28461(56.4)
Less than once a week	2.0	0.2	1.4	11469(13.3)	20.0	18.6	1.1	9388(18.6)
At least once a week	1.2	0.4	0.6	7985(9.2)	16.2	15.4	0.7	8838(17.5)
Almost every day	1.5	0.8	0.3	4036(4.7)	14.0	13.1	0.5	3793(7.5)
***Frequency of listening to radio***								
Not at all	4.5	0.6	2.1	27885(32.3)	22.7	19.2	1.9	9387(18.6)
Less than once a week	5.2	0.4	3.3	14370(16.6)	22.3	20.0	1.4	8277(16.4)
At least once a week	3.4	0.3	1.4	16898(19.6)	22.6	20.7	1.4	14447(28.6)
Almost every day	2.3	0.5	1.4	27280(31.6)	18.9	17.8	0.8	18369(36.4)
***Frequency of watching television***								
Not at all	4.1	0.5	2.0	59568(68.9)	24.2	21.3	1.6	25276(50.1)
Less than once a week	6.0	0.4	4.6	8465(9.8)	20.9	19.6	1.1	9146(18.1)
At least once a week	1.4	0.4	0.7	7298(8.4)	18.7	17.7	1.1	10020(19.8)
Almost every day	1.2	0.5	0.3	11102(12.8)	13.5	12.8	0.3	6038(12.0)
***Age 5-year groups***								
15–19	0.6	0.1	0.1	17642(20.4)	4.2	3.8	0.2	11649(23.1)
20–24	1.6	0.3	0.8	17933(20.7)	16.0	15.1	0.7	8642(17.1)
25–29	2.9	0.4	1.6	15902(18.4)	25.8	24.2	1.2	7334(14.5)
30–34	3.6	0.4	1.9	12106(14.0)	28.9	26.9	1.5	6487(12.9)
35–39	5.7	0.6	2.9	9573(11.1)	31.7	29.4	1.9	5596(11.1)
40–44	8.7	0.9	4.6	7252(8.4)	30.0	26.3	2.3	4173(8.3)
45–49	11.9	1.3	6.8	6025(7.0)	31.6	27.7	2.5	3545(7.0)
50–54					32.4	27.1	3.3	2119(4.2)
55–59					25.5	17.5	1.5	935(1.9)
***Type of place of residence***								
Urban	1.6	0.5	0.6	26201(30.3)	18.2	17.3	0.8	15575(30.9)
Rural	4.6	0.5	2.5	60232(69.7)	22.6	20.1	1.5	34905(69.1)
***Highest educational level***								
None	5.8	0.6	2.3	29681(34.3)	28.0	22.8	2.4	9586(19.0)
Primary	3.6	0.4	2.6	32068(37.1)	23.0	21.5	1.4	19756(39.1)
Secondary and above	1.2	0.4	0.6	24684(28.6)	16.4	15.6	0.6	21138(41.9)
***Religion***								
Christian	3.7	0.5	2.3	64156(74.2)	18.2	16.9	1.0	37418(74.1)
Muslim	2.8	0.3	0.6	18480(21.4)	24.8	21.4	1.7	8172(16.2)
Other	8.3	1.1	2.8	3797(4.4)	38.7	33.9	3.0	4890(9.7)
***Wealth index***								
Poorest	8.5	0.6	5.1	16057(18.6)	29.3	24.3	2.4	8871(17.6)
Poorer	4.2	0.5	2.2	15563(18.0)	24.3	22.1	1.6	9101(18.0)
Middle	3.2	0.5	1.7	15976(18.5)	21.8	20.3	1.2	9549(18.9)
Richer	2.2	0.3	1.1	17084(19.8)	19.0	18.0	0.7	10407(20.6)
Richest	1.2	0.4	0.3	21753(25.2)	14.6	13.9	0.7	12552(24.9)
***Current marital status***								
Not married	0.9	0.3	0.4	20419(23.6)	11.1	10.4	0.4	21309(42.2)
Currently married	4.2	0.5	2.2	57365(66.4)	27.5	24.6	1.9	26970(53.4)
Formerly married	6.4	0.9	4.1	8649(10.0)	42.5	39.4	2.6	2201(4.4)
***Employed***								
no	2.9	0.4	1.6	38801(44.9)	13.5	12.4	0.8	11961(23.7)
yes	4.3	0.5	2.2	47632(55.1)	23.6	21.4	1.4	38519(76.3)
***Level of tobacco bans***								
Fair	4.8	0.4	2.3	43768(50.6)	21.7	18.8	1.5	20813(41.2)
None	2.6	0.6	1.5	42665(49.4)	20.9	19.6	1.1	29667(58.8)
***Country code and phase***								
Burkina Faso	4.0	0.1	0.6	17087(19.8)	23.3	20.3	1.8	7307(14.5)
Ethiopia	3.1	0.5	0.3	14070(16.3)	16.5	12.0	1.1	6033(12.0)
Liberia	2.7	0.9	1.8	7092(8.2)	16.8	15.8	0.4	6009(11.9)
Lesotho	11.1	0.2	10.7	7624(8.8)	33.9	33.9	0.3	3317(6.6)
Malawi	2.1	0.4	0.8	11651(13.5)	18.1	17.7	0.3	7175(14.2)
Swaziland	2.2	1.2	1.0	4987(5.8)	16.7	13.9	2.3	4156(8.2)
Uganda	5.8	0.5	4.1	7878(9.1)	23.9	18.3	6.2	2503(5.0)
Zambia	1.8	0.8	0.9	7142(8.3)	24.1	23.8	0.2	6500(12.9)
Zimbabwe	0.9	0.3	0.5	8902(10.3)	22.9	21.3	1.7	7480(14.8)
**Total**	**3.7**	**0.5**	**1.9**	**86433(100.0)**	21.2	19.3	1.3	50480(100.0)

The results suggest that a mere 3.6% of the women in the region read newspapers or magazines every day and that 71.6% did not read print media material at all. The frequency of watching television in this region was also very low. Less that 9% of the women in the pooled sample indicated that they watched television almost every day and yet another 63.5% indicated that they never watched television at all. The results suggested that radio was the most dominant mass-medium among the 9 SSA countries considered. In the pooled sample, 25.5% of the women indicated that they listened to radio almost every day. In the male sample, 36% of the men listened to radio almost every day, 12% watched television every day and less than 10% read newspapers or magazines every day.

There were significant national variations in tobacco use. In the female sample, tobacco use was highest in Lesotho (11%), followed by Uganda (6%) and Burkina Faso (4%), while Zimbabwe (0.9%) had the lowest use of tobacco. In the male sample, the prevalence of tobacco use was highest in Lesotho (34%), Zambia (24%) and Uganda (24%), and lowest in Ethiopia (17%).

In the female sample, the prevalence of tobacco use and cigarette smoking declined with increasing media utilization. There was a non-linear relationship between snuff use and media utilization. However, the prevalence of snuff use was lowest among those women with the greatest media utilization. The incidence of tobacco use and snuff use was lowest among those women who watched television almost every day. The rate of cigarette smoking was lower in women who watched television almost every day compared to those who watched television at least once a week or less than once a week.

In the male sample, the prevalence of snuff use and overall tobacco use declined with increasing media utilization. The prevalence of cigarette smoking was lowest among those men within the highest media utilization category. There was a significant decline in the prevalence of tobacco use, cigarette smoking and snuff use with increasing frequency of television watching among men. In all three types of tobacco use categories considered, prevalence of use was lowest among men who watched television almost every day.

### Statistical modelling

Table 3 presents the results of generalized linear mixed effects modelling for the female sample. Five separate models were considered. The first model investigated the impact of the media utilization index on tobacco use. Tobacco use among women with high media utilization was 5% lower than that for women with low media utilization. However, there was a marginally significant association between media utilization and tobacco use, adjusting for the selected covariates. Tobacco use was 12% lower for women who listened to radio at least once a week compared to those who never listened to radio at all. The results of this study suggest that the reading of newspaper (or magazines) and the watching of television were not significant association with tobacco use.

**Table 3:** Logistic regression analysis results for the Female population.

Variable	Tobacco use	Smoking	Snuff	Model 1	Model 2	Model 3	Model 4	Model 5
	OR(95%CI)	OR(95%CI)	OR(95%CI)	AOR(95%CI)	AOR(95%CI)	AOR(95%CI)	AOR(95%CI)	AOR(95%CI)
***Fixed effects***								
*Media exposure index*								
Low	1.00(Ref.)	1.00(Ref.)	1.00(Ref.)	1.00(Ref.)				
Medium	0.89**(0.83–0.97)	0.72** (0.58–0.90)	1.12*(1.01–1.25)	1.07(0.97–1.18)				
High	0.34***(0.31–0.38)	0.72** (0.58–0.91)	0.39*** (0.34–0.45)	0.95(0.82–1.00)				
*Frequency of reading newspaper or magazine*								1.00(Ref.)
Not at all	1.00(Ref.)	1.00(Ref.)	1.00(Ref.)		1.00(Ref.)			0.94(0.80–1.11)
Less than once a week	0.42*** (0.37–0.48)	0.65** (0.48–0.89)	0.58*** (0.49–0.68)		0.95(0.81–1.12)			0.88(0.70–1.12)
At least once a week	0.28*** (0.23–0.34)	0.85(0.61–1.18)	0.27*** (0.20–0.35)		0.89(0.71–1.12)			1.09(0.77–1.56)
Almost every day	0.36*** (0.27–0.48)	1.50(1.00–2.25)	0.14*** (0.08–0.27)		1.16(0.82–1.64)			
*Frequency of listening to radio*								
Not at all	1.00(Ref.)	1.00(Ref.)	1.00(Ref.)			1.00(Ref.)		1.00(Ref.)
Less than once a week	0.97(0.88–1.07)	0.67** (0.51–0.88)	1.30*** (1.15–1.48)			0.97(0.86–1.10)		0.97(0.85–1.10)
At least once a week	0.67*** (0.60–0.73)	0.44*** (0.33–0.59)	0.62*** (0.53–0.72)			0.88* (0.78–1.00)		0.88(0.78–1.00)
Almost every day	0.58*** (0.53–0.65)	0.91(0.72–1.14)	0.81** (0.71–0.93)			1.04(0.90–1.19)		1.03(0.90–1.18)
*Frequency of watching television*								
Not at all	1.00(Ref.)	1.00(Ref.)	1.00(Ref.)				1.00(Ref.)	1.00(Ref.)
Less than once a week	1.02(0.92–1.12)	0.73* (0.54–1.00)	1.42*** (1.26–1.60)				1.05(0.91–1.21)	1.07(0.92–1.24)
At least once a week	0.36*** (0.31–0.42)	0.69* (0.51–0.94)	0.23*** (0.18–0.30)				0.96(0.79–1.17)	1.00(0.82–1.22)
Almost every day	0.35*** (0.28–0.42)	1.26(0.93–1.71)	0.21*** (0.15–0.30)				1.23(0.94–1.60)	1.21(0.92–1.58)
*Age in 5-year groups*								
15–19	1.00(Ref.)	1.00(Ref.)	1.00(Ref.)	1.00(Ref.)	1.00(Ref.)	1.00(Ref.)	1.00(Ref.)	1.00(Ref.)
20–24	2.48*** (1.99–3.10)	2.91*** (1.78–4.77)	2.71*** (1.92–3.83)	2.60*** (2.01–3.36)	2.60*** (2.01–3.36)	2.60*** (2.01–3.36)	2.60*** (2.01–3.36)	2.60*** (2.01–3.36)
25–29	4.70*** (3.82–5.78)	3.90*** (2.41–6.30)	5.74*** (4.17–7.90)	4.96*** (3.85–6.40)	4.96*** (3.84–6.39)	4.95*** (3.84–6.39)	4.96*** (3.84–6.39)	4.95*** (3.84–6.38)
30–34	6.12*** (4.98–7.53)	4.94*** (3.05–7.99)	7.11*** (5.16–9.80)	6.58*** (5.08–8.52)	6.56*** (5.07–8.50)	6.54*** (5.05–8.48)	6.55*** (5.05–8.48)	6.53*** (5.04–8.46)
35–39	9.26*** (7.56–11.35)	6.03*** (3.73–9.76)	10.82*** (7.89–14.8)	10.62*** (8.21–13.7)	10.60*** (8.19–13.7)	10.56*** (8.16–13.7)	10.57*** (8.17–13.7)	10.55*** (8.15–13.7)
40–44	16.12*** (13.2–19.7)	8.89*** (5.52–14.31)	20.14*** (14.8–27.4)	19.17*** (14.8–24.8)	19.1*** (14.78–24.7)	19.0*** (14.72–24.6)	19.1*** (14.8–24.67)	19.0*** (14.72–24.62)
45–49	21.38*** (17.5–26.1)	11.98***7.5–19.14)	30.47*** (22.5–41.3)	27.57*** (21.3–35.7)	27.52*** (21.3–35.6)	27.35*** (21.1–35.4)	27.46*** (21.2–35.6)	27.34*** (21.1–35.4)
*Type of place of residence*								
Urban	1.00(Ref.)	1.00(Ref.)	1.00(Ref.)	1.00(Ref.)	1.00(Ref.)	1.00(Ref.)	1.00(Ref.)	1.00(Ref.)
Rural	2.35*** (2.13–2.60)	0.91(0.74–1.11)	3.67*** (3.12–4.32)	0.98(0.79–1.21)	0.98(0.79–1.22)	0.98(0.79–1.21)	0.98(0.79–1.22)	0.97(0.79–1.21)
*Educational level*								
No education	1.00(Ref.)	1.00(Ref.)	1.00(Ref.)	1.00(Ref.)	1.00(Ref.)	1.00(Ref.)	1.00(Ref.)	1.00(Ref.)
Primary	0.52*** (0.48–0.56)	0.61*** (0.49–0.75)	0.98(0.88–1.09)	0.85**(0.75–0.96)	0.85** (0.76–0.96)	0.84** (0.75–0.94)	0.84** (0.75–0.95)	0.85** (0.75–0.96)
Secondary	0.20*** (0.18–0.23)	0.55*** (0.43–0.71)	0.30*** (0.25–0.35)	0.50*** (0.42–0.60)	0.50*** (0.41–0.60)	0.49*** (0.41–0.58)	0.48*** (0.40–0.58)	0.49*** (0.41–0.59)
Higher	0.09*** (0.06–0.15)	0.26** (0.11–0.63)	0.10*** (0.04–0.21)	0.22*** (0.13–0.37)	0.21*** (0.12–0.37)	0.21*** (0.12–0.35)	0.20*** (0.12–0.35)	0.21*** (0.12–0.36)
*Religion*								
Christian	1.00(Ref.)	1.00(Ref.)	1.00(Ref.)	1.00(Ref.)	1.00(Ref.)	1.00(Ref.)	1.00(Ref.)	1.00(Ref.)
Muslim	1.25*** (1.15–1.36)	0.44*** (0.34–0.57)	2.13*** (1.88–2.41)	0.92(0.81–1.04)	0.92(0.81–1.05)	0.93(0.82–1.05)	0.93(0.82–1.05)	0.93(0.82–1.05)
Other	1.03(0.94–1.12)	0.66*** (0.53–0.81)	1.41*** (1.24–1.61)	1.12(0.98–1.27)	1.12(0.98–1.27)	1.12(0.99–1.27)	1.13(0.99–1.28)	1.12(0.99–1.28)
*Wealth index*								
Poorest	1.00(Ref.)	1.00(Ref.)	1.00(Ref.)	1.00(Ref.)	1.00(Ref.)	1.00(Ref.)	1.00(Ref.)	1.00(Ref.)
Poorer	0.51*** (0.46–0.56)	0.89(0.67–1.18)	0.46*** (0.40–0.52)	0.71*** (0.63–0.80)	0.71*** (0.63–0.80)	0.71*** (0.63–0.81)	0.71*** (0.63–0.80)	0.71*** (0.63–0.81)
Middle	0.39*** (0.35–0.43)	0.77(0.57–1.03)	0.35*** (0.30–0.40)	0.61*** (0.53–0.70)	0.61*** (0.53–0.70)	0.62*** (0.54–0.71)	0.61*** (0.53–0.70)	0.62*** (0.54–0.71)
Richer	0.27*** (0.24–0.30)	0.66**(0.49–0.88)	0.22*** (0.19–0.26)	0.45*** (0.39–0.53)	0.45*** (0.39–0.53)	0.45*** (0.39–0.53)	0.45*** (0.38–0.53)	0.45*** (0.39–0.53)
Richest	0.17*** (0.15–0.19)	0.66** (0.50–0.87)	0.07*** (0.06–0.09)	0.28*** (0.23–0.35)	0.28*** (0.22–0.35)	0.28*** (0.22–0.35)	0.27*** (0.21–0.34)	0.27*** (0.21–0.34)
*Current marital status*								
Never married	1.00(Ref.)	1.00(Ref.)	1.00(Ref.)	1.00(Ref.)	1.00(Ref.)	1.00(Ref.)	1.00(Ref.)	1.00(Ref.)
Married	4.63*** (4.01–5.34)	1.82*** (1.38–2.39)	4.51*** (3.68–5.52)	1.05(0.86–1.27)	1.05(0.87–1.28)	1.05(0.87–1.28)	1.06(0.87–1.28)	1.05(0.87–1.28)
Separated	6.89*** (5.87–8.09)	3.63*** (2.63–5.00)	8.09*** (6.48–10.10)	1.37** (1.11–1.70)	1.38** (1.11–1.71)	1.38** (1.11–1.71)	1.38** (1.12–1.71)	1.38** (1.11–1.71)
*Currently working*								
No	1.00(Ref.)	1.00(Ref.)	1.00(Ref.)	1.00(Ref.)	1.00(Ref.)	1.00(Ref.)	1.00(Ref.)	1.00(Ref.)
Yes	1.64*** (1.52–1.76)	1.23* (1.02–1.49)	1.32*** (1.20–1.46)	1.15** (1.04–1.27)	1.15** (1.04–1.27)	1.15** (1.04–1.27)	1.15** (1.04–1.27)	1.15** (1.04–1.28)
*Level of tobacco ban*								
Fair	1.00(Ref.)	1.00(Ref.)	1.00(Ref.)	1.00(Ref.)	1.00(Ref.)	1.00(Ref.)	1.00(Ref.)	1.00(Ref.)
None	2.47*** (2.29–2.66)	0.64*** (0.52–0.78)	2.08*** (1.88–2.31)	0.27*** (0.23–0.31)	0.27*** (0.23–0.32)	0.26*** (0.22–0.31)	0.27*** (0.23–0.31)	0.26*** (0.22–0.31)
								
***Random effects***								
Cluster variance				2.95(2.63–3.30)	2.95(2.64–3.30)	2.94(2.63–3.29)	2.94(2.63–3.29)	2.92(2.61–3.27)
No. of observations	101 316			101 316	101 316	101 316	101 316	101 316
No. of clusters	4 119			4 119	4 119	4 119	4 119	4 119

* p<0.05

** p<0.01

*** p<0.001.

The results also suggested that tobacco use among women increased with increasing age and was higher in urban areas compared to rural areas. There was also a decline in tobacco use with increasing levels of education and socioeconomic status. Formerly married women were more likely to use tobacco compared to women who had never married. However, there was no significant difference in tobacco use between currently married women and women that had never married. There was no association between religion and tobacco use, after adjusting for the effect of other covariates. Tobacco use in countries where the FCTC recommendations had been implemented was found to be higher than in countries that had not adopted the FCTC recommendations.

Table 4 presents logistic regression results for the male sample. The results (adjusting for the effects of other covariates) suggest that tobacco use declined with increased media utilization as seen in the frequency of reading newspapers or magazines and watching television.

**Table 4:** Logistic regression analysis results for the Male population.

	Tobacco use	Smoking	Snuff	Model 1	Model 2	Model 3	Model 4	Model 5
	OR(95%CI)	OR(95%CI)	OR(95%CI)	AOR(95%CI)	AOR(95%CI)	AOR(95%CI)	AOR(95%CI)	AOR(95%CI)
***Fixed Effects***								
*Media exposure index*								
Low	1.00(Ref.)	1.00(Ref.)	1.00(Ref.)	1.00(Ref.)				
Medium	0.90*** (0.86–0.95)	1.02(0.97–1.08)	0.75**(0.63–0.89)	1.00(0.94–1.06)				
High	0.59*** (0.56–0.62)	0.69*** (0.66–0.73)	0.32*** (0.26–0.41)	0.79(0.73–0.85)				
*Frequency of reading newspaper or magazine*								1.00(Ref.)
Not at all	1.00(Ref.)	1.00(Ref.)	1.00(Ref.)		1.00(Ref.)			0.94(0.88–1.01)
Less than once a week	0.77*** (0.73–0.81)	0.83*** (0.79–0.88)	0.49*** (0.39–0.62)		0.95(0.88–1.01)			0.81(0.75–0.87)
At least once a week	0.62*** (0.59–0.66)	0.69*** (0.65–0.73)	0.40*** (0.31–0.52)		0.80(0.74–0.87)			0.71(0.62–0.81)
Almost every day	0.53*** (0.48–0.59)	0.59*** (0.53–0.66)	0.34*** (0.21–0.54)		0.65(0.57–0.74)			
*Frequency of listening to radio*								
Not at all	1.00(Ref.)	1.00(Ref.)	1.00(Ref.)			1.00(Ref.)		1.00(Ref.)
Less than once a week	1.00(0.93–1.06)	1.03(0.96–1.10)	0.89(0.70–1.12)			1.11(1.03–1.20)		1.12(1.04–1.21)
At least once a week	0.99(0.93–1.05)	1.06(0.99–1.12)	0.79*(0.64–0.97)			1.08(1.01–1.16)		1.10(1.02–1.18)
Almost every day	0.89*** (0.84–0.95)	1.02(0.95–1.08)	0.53*** (0.42–0.67)			0.88(0.81–0.95)		0.92(0.85–1.00)
*Frequency of watching television*								
Not at all	1.00(Ref.)	1.00(Ref.)	1.00(Ref.)				1.00(Ref.)	1.00(Ref.)
Less than once a week	0.78*** (0.74–0.82)	0.81*** (0.77–0.86)	0.77**(0.63–0.94)				0.99(0.93–1.05)	0.98(0.92–1.05)
At least once a week	0.69*** (0.65–0.72)	0.75*** (0.71–0.79)	0.55*** (0.45–0.68)				1.00(0.93–1.07)	1.02(0.95–1.10)
Almost every day	0.48*** (0.44–0.52)	0.54*** (0.49–0.59)	0.20*** (0.12–0.33)				0.70(0.62–0.78)	0.75(0.67–0.85)
*Age in 5-year groups*								
15–19	1.00(Ref.)	1.00(Ref.)	1.00(Ref.)	1.00(Ref.)	1.00(Ref.)	1.00(Ref.)	1.00(Ref.)	1.00(Ref.)
20–24	4.18*** (3.77–4.63)	4.27*** (3.8–4.76)	3.07*** (1.90–4.96)	4.73(4.23–5.28)	4.72(4.22–5.27)	4.70(4.21–5.25)	4.69(4.19–5.23)	4.71(4.21–5.26)
25–29	7.36*** (6.67–8.14)	7.37*** (6.6–8.17)	5.86*** (3.74–9.19)	8.81(7.83–9.91)	8.81(7.83–9.92)	8.79(7.81–9.89)	8.69(7.72–9.77)	8.77(7.80–9.87)
30–34	8.99*** (8.13–9.95)	8.90*** (8.0–9.88)	7.63*** (4.88–11.91)	10.40(9.2–11.8)	10.43(9.2–11.82)	10.41(9.18–11.8)	10.28(9.1–11.7)	10.40(9.18–11.79)
35–39	9.7*** (8.7–10.7)	9.4*** (8.46–10.5)	8.49*** (5.4–13.3)	11.6(10.2–13.2)	11.(10.3–13.29)	11.6(10.2–13.2)	11.5(10.1–13.1)	11.65(10.24–13.25)
40–44	9.55*** (8.59–10.6)	8.83*** (7.9–9.87)	10.0*** (6.38–15.79)	11.5(10.1–13.2)	11.6(10.1–13.25)	11.6(10.10–13.21)	11.35(9.9–13.0)	11.56(10.11–13.23)
45–49	10.15*** (9.1–11.32)	9.16*** (8.2–10.27)	12.8*** (8.15–20.05)	12.0(10.49–13.8)	12.1(10.57–13.91)	12.1(10.53–13.85)	11.9(10.35–13.6)	12.08(10.5–13.86)
50–54	10.4*** (9.25–11.8)	8.85*** (7.8–10.1)	15.58*** (9.77–24.8)	11.6(10.0–13.4)	11.6(10.0–13.5)	11.6(10.0–13.5)	11.4(9.8–13.2)	11.65(10.03–13.53)
55–59	7.20*** (6.08–8.53)	4.38*** (3.6–5.33)	12.06*** (6.7–21.6)	7.60(6.22–9.29)	7.65(6.26–9.35)	7.62(6.24–9.32)	7.50(6.14–9.17)	7.66(6.27–9.37)
*Type of place of residence*								
Urban	1.00(Ref.)	1.00(Ref.)	1.00(Ref.)	1.00(Ref.)	1.00(Ref.)	1.00(Ref.)	1.00(Ref.)	1.00(Ref.)
Rural	1.23*** (1.18–1.29)	1.13*** (1.08–1.18)	1.98*** (1.62–2.41)	0.70(0.64–0.77)	0.70(0.64–0.77)	0.73(0.66–0.79)	0.72(0.66–0.79)	0.71(0.65–0.78)
*Educational level*								
No education	1.00(Ref.)	1.00(Ref.)	1.00(Ref.)	1.00(Ref.)	1.00(Ref.)	1.00(Ref.)	1.00(Ref.)	1.00(Ref.)
Primary	0.72*** (0.69–0.76)	0.90*** (0.86–0.96)	0.42*** (0.35–0.50)	1.00(0.94–1.08)	1.01(0.94–1.08)	0.98(0.91–1.05)	0.97(0.91–1.04)	1.00(0.93–1.07)
Secondary	**0.56** *** **(0.53–0.60)**	0.72*** (0.68–0.76)	0.27*** (0.22–0.33)	0.75(0.68–0.81)	0.76(0.70–0.84)	0.70(0.64–0.76)	0.70(0.64–0.76)	0.76(0.70–0.83)
Higher	0.38*** (0.34–0.43)	0.48*** (0.43–0.54)	0.18*** (0.10–0.31)	0.46(0.40–0.54)	0.48(0.41–0.56)	0.42(0.36–0.49)	0.43(0.37–0.50)	0.48(0.42–0.56)
*Religion*								
Christian	1.00(Ref.)	1.00(Ref.)	1.00(Ref.)	1.00(Ref.)	1.00(Ref.)	1.00(Ref.)	1.00(Ref.)	1.00(Ref.)
Muslim	1.13*** (1.07–1.19)	1.13*** (1.07–1.19)	1.42*** (1.17–1.73)	1.26(1.17–1.35)	1.25(1.17–1.34)	1.26(1.18–1.35)	1.26(1.17–1.35)	1.25(1.16–1.34)
Other	1.52*** (1.45–1.59)	1.42*** (1.4–1.5)	1.40*** (1.16–1.70)	1.47(1.37–1.57)	1.46(1.36–1.57)	1.47(1.37–1.58)	1.48(1.37–1.58)	1.46(1.36–1.57)
*Wealth index*								
Poorest	1.00(Ref.)	1.00(Ref.)	1.00(Ref.)	1.00(Ref.)	1.00(Ref.)	1.00(Ref.)	1.00(Ref.)	1.00(Ref.)
Poorer	0.74*** (0.70–0.79)	0.87*** (0.8–0.93)	0.52*** (0.42–0.64)	0.76(0.70–0.82)	0.75(0.70–0.81)	0.76(0.70–0.82)	0.75(0.70–0.81)	0.76(0.70–0.82)
Middle	0.65*** (0.61–0.69)	0.78*** (0.7–0.8)	0.35*** (0.28–0.45)	0.65(0.61–0.71)	0.65(0.60–0.70)	0.65(0.60–0.71)	0.65(0.60–0.70)	0.66(0.61–0.71)
Richer	0.57*** (0.54–0.61)	0.71*** (0.7–0.8)	0.26***(0.20–0.34)	0.55(0.50–0.60)	0.54(0.50–0.59)	0.54(0.50–0.59)	0.54(0.49–0.59)	0.55(0.51–0.60)
Richest	0.45***(0.42–0.48)	0.56***(0.5–0.59)	0.24***(0.19–0.31)	0.44(0.40–0.49)	0.43(0.39–0.48)	0.42(0.38–0.47)	0.44(0.40–0.49)	0.46(0.41–0.51)
*Current marital status*								
Never married	1.00(Ref.)	1.00(Ref.)	1.00(Ref.)	1.00(Ref.)	1.00(Ref.)	1.00(Ref.)	1.00(Ref.)	1.00(Ref.)
Married	2.93***(2.79–3.07)	2.70***(2.57–2.8)	4.68***(3.72–5.88)	0.94(0.87–1.01)	0.94(0.87–1.01)	0.94(0.88–1.02)	0.94(0.87–1.01)	0.94(0.88–1.02)
Separated	5.72***(5.22–6.26)	5.38***(4.9–5.90)	5.91***(4.16–8.40)	1.94(1.74–2.17)	1.94(1.74–2.17)	1.94(1.74–2.17)	1.94(1.73–2.16)	1.94(1.73–2.16)
*Currently working*								
No	1.00(Ref.)	1.00(Ref.)	1.00(Ref.)	1.00(Ref.)	1.00(Ref.)	1.00(Ref.)	1.00(Ref.)	1.00(Ref.)
Yes	1.69***(1.61–1.78)	1.68***(1.6–1.78)	1.28*(1.05–1.55)	1.10(1.03–1.18)	1.11(1.03–1.18)	1.10(1.03–1.18)	1.10(1.03–1.18)	1.11(1.04–1.18)
*Level of tobacco ban*								
Fair	1.00(Ref.)	1.00(Ref.)	1.00(Ref.)	1.00(Ref.)	1.00(Ref.)	1.00(Ref.)	1.00(Ref.)	1.00(Ref.)
None	0.96*(0.92–1.00)	0.85***(0.8–0.88)	1.36***(1.16–1.59)	0.91(0.55–1.52)	0.90(0.54–1.49)	0.94(0.55–1.60)	0.92(0.54–1.55)	0.93(0.54–1.61)
***Random effects***								
Cluster variance				0.522	0.51814	0.51653	0.51591	0.51519
Country variance				0.147	0.14724	0.15939	0.1555	0.16757
Number of observations				58146.00	58146.00	58146.00	58146.00	58146.00
Number of clusters				4117.00	4117.00	4117.00	4117.00	4117.00
Number of countries				9.00	9.00	9.00	9.00	9.00

* p<0.05

** p<0.01

*** p<0.0.

For the male population, tobacco use increased with age and was significantly higher in urban areas. Tobacco use also declined significantly with increasing levels of education and socioeconomic status. There were significant religious variations in tobacco use. Men of the Islamic faith had a 20% higher risk of using tobacco compared to those of the Christian faith. The results of this study further suggest that there is no significant difference in tobacco use between men in countries that have implemented the FCTC recommendations and those that had not.

In addition, tobacco use was seen to be 15% and 10% higher among women and men, respectively, in formal employment compared to those that were not employed.

## Discussion

In this study, we used data from Demographic and Health surveys conducted in 9 SSA countries to investigate the relationship between tobacco use and media utilization. Mass media messages are, in general, on the rise in SSA, through increased use and access to television and radio, movies, newspapers and magazines, the internet and books, brochures and posters [7]. Media can be viewed as a powerful socialising force as it shapes views on what is acceptable and attractive in our modern society [36]. Our results suggested that a very low percentage of women aged 15–49 years in the SSA region access or utilize mass media facilities on a daily basis. Radio, as suggested elsewhere in the literature [50], is still the dominant mass-medium in the region with the widest geographical reach and greatest audience compared with television, newspapers and other information and communication technologies (ICTs). The renaissance enjoyed by radio has been attributed to greater democratisation in the region, market liberalisation and more affordable technologies [50].

We found a positive association between cigarette smoking and media utilization. This result concurs with others in the literature [35]. We also found a decline in the use of smokeless tobacco with increased mass media use, a finding similar, to a great extent, to others in literature [51].

Current literature suggests that the tobacco epidemic has now expanded to, and become more concentrated in, the world's low and middle-income countries (LMIC). This is partly due to the expansion of the multinational tobacco industry's marketing efforts in Eastern Europe, Asia, Africa, and Latin America [52]. Despite the low prevalence rates in these regions, there is need to draw lessons from the negative experiences associated with tobacco use in high-income nations, so as avoid a repeat in LMICs.

Our study concurs with others in the literature that suggest that the prevalence of cigarette smoking by women in Africa is generally very low [14] and that the prevalence of use of other forms of tobacco is slightly higher but also very low. In line with other studies, women in urban areas are more likely to use cigarettes and less likely to use other tobacco products than women in rural areas [14]. As found in other studies, this study also noted that education lowers the use of other forms of tobacco but less clearly lowers cigarette use. This study did not find any association between religion and any of the forms of tobacco use.

### Strengths and Limitations

In this study, information on the frequency of radio, newspaper/magazine and television use were based on self-reported responses. The same also held for the responses given on the smoking of cigarettes, pipes, chewing of tobacco and use of snuff. These could potentially be affected by recall bias and by the tendency of respondents to give socially-desirable answers. The study design was cross-sectional leading to conclusions of association as opposed to causality in most cases. The data set used in this study was collected over the period 2004–2010 and it is possible that a lot has changed since that period. The results, however, suggest strong correlations that could be used to inform policy.

The finding regarding the impact of FCTC implementation on tobacco simply suggests that implementing countries had a greater burden of tobacco use. To correctly assess the impact of FCTC implementation on tobacco use, a longitudinal (as opposed to a cross-sectional) study would be needed.

This study to a large extent utilized data on the respondent’s access to mass media. Future research needs to capture both the access and content dimensions of media utilization to better understand smoking patterns/behaviours and social influence. This will inform context related anti-smoking campaigns and interventions in SSA.

## Conclusions

In this study, we found a positive correlation between cigarette smoking and media exposure. There was, however, an important inverse relationship between media utilisation and the use of other forms of tobacco. This suggests a need to design tobacco control measures that target the advertising, promotion and sponsorship of all forms of tobacco use, in addition to current strategies that target smoking.

Overall, this study has strengthened the evidence that mass media campaigns, conducted in the context of comprehensive tobacco control programmes, can reduce the prevalence of tobacco smoking, but that campaign reach, intensity, duration and message type may influence success.
